# Effect of Toothbrush Bristle Stiffness and Brushing Force on Cleaning Efficacy

**DOI:** 10.3290/j.ohpd.b4100897

**Published:** 2023-05-17

**Authors:** Moritz Tanner, Raphael Singh, Leonardo Svellenti, Blend Hamza, Thomas Attin, Florian J. Wegehaupt

**Affiliations:** a Resident, Clinic of Conservative and Preventive Dentistry, Center of Dental Medicine, University of Zürich, Zürich, Switzerland. Wrote and reviewed the manuscript.; b Resident, Clinic of General, Special Care and Geriatic Dentistry, Center of Dental Medicine, University of Zürich, Zürich, Switzerland. Performed the experiment, reviewed the manuscript.; c Resident, Department of Periodontology, Endodontology and Cariology, University Center for Dental Medicine, University of Basel, Basel, Switzerland. Critically evaluated and reviewed the manuscript.; d Resident, Clinic of Orthodontics and Pediatric Dentistry, Center of Dental Medicine, University of Zürich, Zürich, Switzerland. Critically evaluated and reviewed the manuscript.; e Professor and Head, Clinic of Conservative and Preventive Dentistry, Center of Dental Medicine, University of Zürich, Zürich, Switzerland. Conceived and designed the experiment, critically evaluated and reviewed the manuscript.; f Assistant Professor and Head, Division of Preventive Dentistry and Oral Epidemiology, Clinic of Conservative and Preventive Dentistry, Center of Dental Medicine, University of Zürich, Zürich, Switzerland. Conceived and designed the experiment, critically evaluated and reviewed the manuscript.

**Keywords:** bristle stiffness, brushing force, cleaning efficacy, toothbrush

## Abstract

**Purpose:**

This study investigated the effect of toothbrush bristle stiffness and brushing force on the cleaning efficacy in vitro.

**Materials and Methods:**

Eighty bovine dentin samples were allocated to eight groups (n=10). Two custom-made toothbrushes of different bristle stiffness (soft and medium) were tested at four different brushing forces (1, 2, 3 and 4 N). Dentin samples were stained in black tea and brushed (60 strokes/min) for a total of 25 min in a brushing machine with an abrasive solution (RDA 67). Photographs were taken after 2 and 25 min of brushing time. Cleaning efficacy was measured planimetrically.

**Results:**

After 2 min of brushing, the soft-bristle toothbrush did not cause statistically significantly different cleaning efficacy at different brushing forces, while the medium-bristle toothbrush cleaned statistically significantly less efficaceously only at 1 N. Comparing the two different toothbrushes, higher cleaning efficacy was observed only at 1 N for the soft-bristle brush. At 25 min brushing time, the soft-bristle cleaned statistically significantly better at 4 N compared to 1 N and 2 N and at 3 N compared to 1 N. Using the medium-bristle, cleaning efficacy increased with increasing brushing force. After 25 min of brushing, no statistically significant difference was observed between the two different toothbrushes.

**Conclusion:**

Irrespective the brushing force, the use of a soft or medium toothbrush results in comparable cleaning efficacy. At 2 min brushing time, increasing the brushing force does not increase the cleaning efficacy.

In Western societies, a great cultural demand for straight, white teeth has existed for decades. As we are usually all born with relatively white teeth – deciduous teeth being even whiter than permanent ones – bright teeth are regarded as a sign of youth, health and cleanliness. In fact, a newly developed oral health-related quality of life (OHRQoL) questionnaire tailored to young adults has revealed tooth colour to be the most important concern of this population.^9^ Even though a number of studies showed tooth colour did not necessarily have an impact on the judgment of facial attractiveness by others,^17,28^ it was reported to be an important motivator for adolescents for daily toothbrushing, especially among smokers^16^ and those who brush their teeth in the morning.^42^

Toothbrushing has repeatedly been shown to be critically important for the prevention of the most common oral diseases, e.g. decay, gingivitis and consequently periodontitis, by mechanical plaque control.^4,7,26,33,36,53,61,66^ However, besides its undoubted significance in maintaining good oral health, it can also be seen as a simple measure for treating extrinsic tooth staining.^10,12^

Extrinsic tooth discolouration is described as the deposition and incorporation of chromogenic compounds on the surface of the tooth or pellicle.^54,55^ Among other sources, colouring substances may be present in everyday beverages such as coffee, tea or red wine, but can also derive from mouthrinses containing chlorhexidine.^1-3,30^ Although not seen to have a disease value, discoloured teeth are perceived by many patients as aesthetically disturbing. Standard treatment options are bleaching or professional tooth cleaning. While bleaching agents – such as hydrogen or carbamide peroxides or sodium perborate – achieve their goal through diffusion into the dental hard tissue and interaction with stain molecules and dental hard tissue,^6,34,41^ professional tooth cleaning using abrasive pastes or powder-jets remove not only plaque and calculus, but also parts of the discoloured tooth surface.^43^ It can be assumed that this is also observed to a certain extent when brushing teeth. As mentioned above, to some people this even seems to be one of the main reasons for brushing their teeth on a daily basis.

The interaction between toothbrushing and its cleaning effect on dental hard tissue has been examined in several studies, most of them showing a positive correlation between abrasive dental hard-tissue wear and cleaning efficacy.^23,31,48^ This can be explained causally by the mechanical removal of the outermost stained layer of dental hard tissue. Several different factors affecting the abrasive wear – and thus the cleaning efficacy on enamel and dentin – have been identified, such as type of toothbrush used, brushing force,^59^ duration and frequency of brushing, brushing technique, or the addition of toothpaste.^11,40,60,62^ Overall, the toothpaste seems to have the biggest impact on abrasive wear, while being modified by other factors such as the ones mentioned above.^4,61^ The abrasivity of toothpastes is specified by the RDA (relative dentin abrasivity) and REA (relative enamel abrasivity).^15^ Higher RDA and REA values indicate higher dentin and enamel wear. Generally, good correlation between dentin wear and RDA values has been shown.^40,50^ However, abrasive wear has been found not to be the only determinant of cleaning efficacy. In a recent study by Hamza et al,^23^ toothpastes containing additives such as diamond powder or charcoal offered high cleaning efficacies with low dentin abrasivity.

As mentioned before, besides the toothpaste itself, some other factors also have an impact on both dentin wear and cleaning efficacy. One of these is the type of toothbrush used, which can vary in terms of stiffness,^37,52,60,62^ arrangement^5,20^ and end configuration of bristles,^22^ to name a few. Numerous studies have investigated their impact on abrasive dental wear as well as their efficacy in removing plaque,^35,65^ showing partially contradictory results. This may be due to non-uniform conditions both in the laboratory setting as well as toothbrush specifications. The classification of toothbrush bristle stiffness is based on the resistance of the tufted portion to deflection, normalised to the surface area of the brushing head. The test procedure is described in ISO standards 8627 and 22254.^38^ Depending solely on their brushing resistance measured in a standardised, automated brushing machine, toothbrushes are classified as soft, medium and hard. However, this disregards other specifications, such as arrangement, number, material, thickness or end configuration of bristles. Because numerous previous studies tested toothbrushes of different specifications, a direct comparison between them hardly seems legitimate. Consequently, there is need for isolated examination of the different features of toothbrushes and their impact on both the dental wear and the cleaning efficacy.

Another recent study by Hamza et al^24^ investigated the effect of toothbrush bristle stiffness and brushing force on the abrasive dentin wear with toothbrushes that differed only in the length of their bristles (longer bristles classified as soft bristles, while shorter bristles are classified as medium hard). For both soft- and medium-bristle toothbrushes, no statistically significantly differences in dentin wear could be shown at 1 N, 2 N and 3 N of applied brushing force. While the medium-bristle toothbrush caused higher dentin wear with increasing brushing force, the soft-bristle toothbrush seemed to reach a peak at 3 N and caused statistically significantly less dentin wear at 4 N than at 3 N.

However, their effect on the resulting cleaning efficacy has not yet been examined. Therefore, the aim of this study was to add another facette to the current research by investigating the effect of toothbrush bristle stiffness and the applied brushing force on the cleaning efficacy. The null hypothesis of the present study was that there is no difference in the cleaning efficacy of soft and medium toothbrushes at each applied brushing force.

## Materials and Methods

### Sample Preparation

Eighty bovine dentin samples harvested from mandibular incisors were randomly divided into eight groups (G1 – G8) (n = 10). Teeth were cleaned of adhering soft tissues using dental scalers and nylon brushes, then crowns were removed with an electric cutting machine (Planopol-2, Struers; Ballerup, Denmark), leaving the roots with a final length of 12 to 15 mm. The most planar parts of the roots were identified and samples were thinned from the back down to a final thickness of approximately 6 mm by using a dental handpiece, while making sure not to open the root canals. Surfaces were polished manually with Sof-Lex PopOn disks (Nr. 1982SF 15” followed by Nr. 2382SF, 3M Schweiz; Rüschlikon, Switzerland) for 60 s under constant water cooling. Rotational speed was set at 1500 RPM and the applied load was set at 40 – 60 g using a pressure gauge (Tetronix 503; Portland, OR, USA). The root canals were sealed with modelling clay (Pelikan Plastilin weiss, Pelikan; Schindellegi, Switzerland) and nail varnish (essence shine last & go, Cosnova; Sulzbach, Germany) to prevent internal staining.

**Fig 1:**
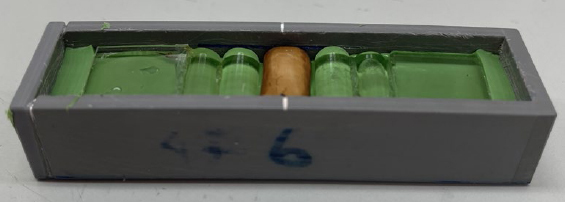
Example of an embedded dentin sample.

Samples were then stained in a black tea solution, for which one teabag of Lipton Yellow Label Tea Quality No. 1 (Unilever; London, UK) and one teabag of EXTRA STRONG TEABAGS (Marks & Spencer; Lancing, UK) were put into 390 ml of boiling deionised water (TKA MicroPure, TKA Wasseraufbereitungssysteme; Niederelbert, Germany) for 10 min. After cooling, the tea solution was filled up to 400 ml with deionised water and the pH was adjusted to 4 by drop-wise addition of citric acid (780 pH Meter, Metrohm Schweiz; Zofingen, Switzerland). Samples were subjected to staining for 17 h at 40°C in a drying cabinet (Serie FD Classic Line, Binder; Tuttlingen, Germany) under gentle movement on an orbital shaker (IKA Vibrax VXR, IKA-Werke; Staufen, Germany). Samples were then rinsed with tap water and embedded in an impression material (President Plus Light Body, Coltene Whaledent; Altstätten, Switzerland) in the center of a plastic container with the selected surface facing up, with two cylinders placed on each side and one cuboid made of glass at both ends of the container (Fig 1).

### Toothbrushes

**Fig 2:**
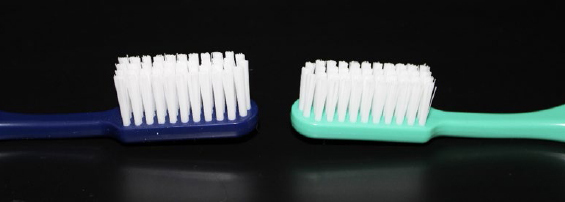
Toothbrushes used in this study, with 12 mm of bristle length (‘soft’, left side) and 10.5 mm of bristle length (‘medium’, right side).

The toothbrushes used were custom-made (Paro M43, Esro; Kilchberg, Switzerland) and differed only in the length of their bristles, which defined their property ‘soft’ or ‘medium’ (Table 1 and Fig 2).

**Fig 3:**
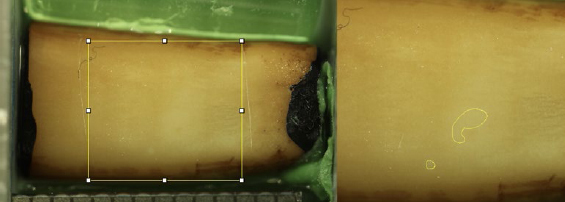
Example of ROI with corresponding pixel count after 2 min of brushing with medium-bristle toothbrush at 1 N of brushing force.

**Fig 4:**
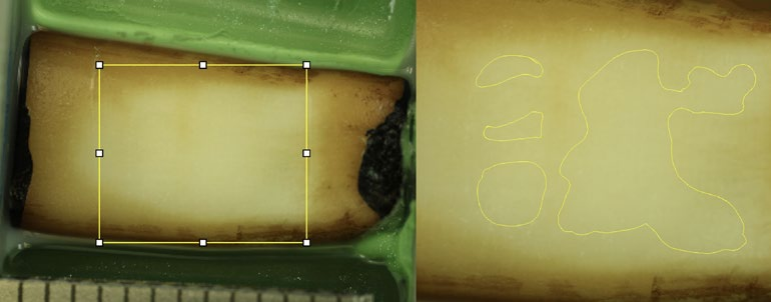
Example of ROI with corresponding pixel count after 25 min of brushing with medium-bristle toothbrush at 1 N of brushing force.

**Table 1:** Specifications of toothbrushes used (according to manufacturer’s information)

Parameter	Soft	Medium
Bristle diameter	0.2 mm	0.2 mm
Bristle length	12 ± 0.2 mm	10.5 ± 0.2 mm
Material	Polyamide	Polyamide
Tip configuration	Rounded end	Rounded end
Number of tufts	43	43
Number of bristles per tuft	40 ± 4	40 ± 4

### Brushing Process

Six containers at a time were screwed onto an automated brushing machine (custom-made by the lab of the Clinic of Conservative and Preventive Dentistry, University of Zürich) perpendicular to the brushing direction. The respective toothbrush heads were fixed to the brushing machine. The areas to be brushed of the stained roots were marked with scratches using a scalpel, and baseline photos were taken using a digital camera (EOS 2000D, Canon; Tokyo, Japan) and a magnifying lens (Macro Photo Lens MP-E 65 mm, Canon).

Samples were then brushed with the corresponding toothbrush (groups 1 to 4: soft; groups 5 to 8: medium) and brushing force (groups 1 and 5: 1 N; groups 2 and 6: 2 N; groups 3 and 7: 3 N; groups 4 and 8: 4 N) for a total of 25 min using an abrasive slurry with an RDA of 67.^29^ The slurry was prepared according to a lab-internal recipe by mixing 90 g of silicate powder (Zeodent 113, Evonik Industries; Hanau-Wolfgang, Germany), 450 g of glycerine and 0.45 g of silicone antifoam agent (Sigma-Aldrich; St Louis, MO, USA). Brushing speed was set at 60 strokes/min, and 1 ml of abrasive slurry was added to each container for every brushing sequence. After 2, 5 and 10 min, the brushing sequence was stopped, the samples were rinsed with tap water and dried with paper tissue, after which new photos were taken using the same settings as at baseline. Between brushing sequences, the brushing force was repeatedly checked using a pressure gauge (PESOLA 600 g, PESOLA Präzisionswaagen; Schindellegi, Switzerland).

### Evaluation of Cleaning Efficacy

Pre- and post-brushing photos were then analysed planimetrically by a single investigator (RS) using the application ‘Fiji’ based on the open-source platform ImageJ for biological image analysis^45^ on a tablet computer (Surface Pro 7, 12.3-inch, Microsoft; Redmond, WA, USA). The ROI (region of interest) was set as a rectangle defined horizontally by the scratches made on the samples prior to brushing and vertically by the outermost lines completely located on dentin. The ROI surface area was recorded in pixels; a subjectively-perceived stain-free surface area was also captured and recorded in pixels using a pen (Surface Pen Stylus, Microsoft). The cleaning efficacy in percent was then obtained by dividing the number of pixels of the stain-free area by the number of pixels from the corresponding ROI, multiplied by 100.

### Statistical Analysis

Data were collected using Microsoft Excel and statistical testing was conducted using the language and environment for statistical computing ‘R’ (The R Foundation For Statistical Computing; Vienna, Austria).^49,58^ Statistical analysis of the cleaning efficacy was performed separately for the 2 min and 25 min brushing times. To investigate differences between applied brushing forces for the same bristle stiffness, Kruskal-Wallis test was conducted. Pairwise Conover post-hoc tests were applied between these different brushing forces, and p-values were adjusted following the Holm method for multiple testing. Finally, comparisons in the cleaning efficacy at same brushing forces for the two different bristle stiffnesses were tested with the Wilcoxon rank-sum test, and p-values were again adjusted following the Holm method.

## Results

The datasets generated and/or analysed during the current study are available from the corresponding author on reasonable request.

**Fig 5:**
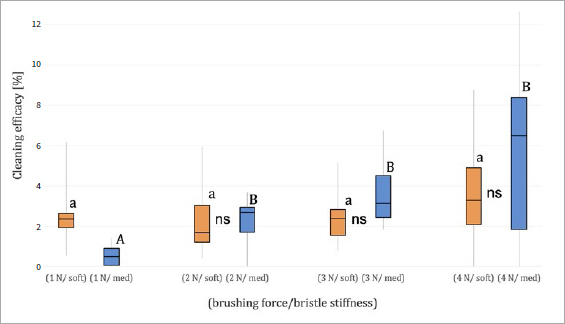
Median, quartiles, minimum and maximum of cleaning efficacy [%] of different bristle stiffness (soft and medium) at different brushing forces (1 N, 2 N, 3 N, 4 N) after 2 min. Values within the same bristle stiffness for the different brushing forces that do not differ statistically significantly are marked with same letters (lower case letters for soft, capital letters for medium). Values within the same brushing force for the different bristle stiffnesses that do not differ statistically significantly are marked as ‘ns’.

The cleaning efficacy (%) for the two different bristle stiffnesses (soft and medium) and the four brushing forces (1 N, 2 N, 3 N and 4 N) after 2 min brushing time are presented in Table 2 and Fig 5.

**Table 2:** Descriptive statistics after 2 min of brushing

Bristle stiffness	Brushing force [N]	Mean cleaning efficacy	SD cleaning efficacy	Median cleaning efficacy	IQR cleaning efficacy	Min cleaning efficacy	Max cleaning efficacy
Soft	1	2.44	1.48	2.36	0.71	0.55	6.16
Medium	1	0.54	0.50	0.50	0.84	0.00	1.41
Soft	2	2.37	1.95	1.68	1.84	0.42	5.94
Medium	2	2.35	1.11	2.68	1.25	0.00	3.68
Soft	3	2.39	1.31	2.37	1.28	0.78	5.14
Medium	3	3.70	1.78	3.13	2.08	1.84	6.74
Soft	4	3.96	2.81	3.28	2.82	0.00	8.77
Medium	4	5.92	4.54	6.50	6.54	0.00	12.63

Values for mean, standard deviation (SD), median, interquartile range (IQR), minimum (min) and maximum (max) cleaning efficacy are given in % of the ROI.

At 2 min brushing time with the soft-bristle toothbrush, increasing the brushing force from 1 N through to 4 N did not provide a statistically significant improvement in the cleaning efficacy (p > 0.05). In contrast, brushing with the medium-bristle toothbrush yielded statistically significantly better cleaning efficacy at >1 N brushing force (p < 0.05). Comparing the two different toothbrushes, statistically significantly higher cleaning efficacy was observed only at 1 N brushing force for the soft-bristle toothbrush (p < 0.05).

**Fig 6:**
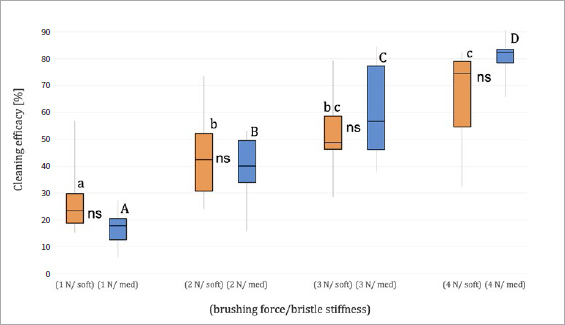
Median, quartiles, minimum and maximum of cleaning efficacy [%] of different bristle stiffness (soft and medium) at different brushing forces (1 N, 2 N, 3 N, 4 N) after 25 min. Values within the same bristle stiffness for the different brushing forces that do not differ statistically significantly are marked with same letters (lower case letters for soft, capital letters for medium). Values within the same brushing force for the different bristle stiffnesses that do not differ statistically significantly are marked as ‘ns’.

The cleaning efficacy (%) of the two different bristle stiffnesses (soft and medium) and the four brushing forces (1 N, 2 N, 3 N and 4 N) after 25 min brushing time are presented in Table 3 and Fig 6.

**Table 3:** Descriptive statistics after 25 min of brushing

Bristle stiffness	Brushing force [N]	Mean cleaning efficacy	SD cleaning efficacy	Median cleaning efficacy	IQR cleaning efficacy	Min cleaning efficacy	Max cleaning efficacy
Soft	1	27.4	13.15	23.4	11.00	15.23	57.0
Medium	1	17.1	6.85	17.9	7.92	6.23	27.2
Soft	2	43.1	15.64	42.4	21.44	24.14	73.6
Medium	2	39.1	12.39	40.1	15.64	15.77	53.2
Soft	3	50.6	15.08	48.8	12.37	28.69	79.4
Medium	3	60.8	17.73	56.8	31.04	37.81	84.7
Soft	4	65.5	18.18	74.6	24.40	32.44	82.7
Medium	4	80.5	7.90	82.5	5.02	65.80	90.7

Values for mean, standard deviation (SD), median, interquartile range (IQR), minimum (min) and maximum (max) cleaning efficacy are stated in % of ROI.

After 25 min of brushing, the soft-bristle toothbrush yielded statistically significantly higher cleaning efficacy at 4 N than at 1 N and 2 N (p < 0.05) and at 3 N compared to 1 N (p < 0.05). Using the medium-bristle toothbrush, cleaning efficacy increased gradually and statistically significantly with increasing brushing force (p < 0.05). However, no statistically significant difference in cleaning efficacy was observed between the two different toothbrushes at the respective brushing forces (p > 0.05).

## Discussion

Toothbrushing is an integral part of everyday life. Customers can choose from a continuously increasing variety of toothbrushes with different properties, including bristle stiffness. Despite its undoubted benefit for preserving oral health by means of mechanical plaque control, the desire for white teeth is also an important motivator for daily toothbrushing. Therefore, this study investigated the effect of toothbrush bristle stiffness and brushing force on cleaning efficacy. The null hypothesis – that there is no difference in the cleaning efficacy of soft and medium toothbrushes at each applied brushing force – had to be rejected.

In this study, bovine dentin was used as a substitute for human dentin. The use of bovine root dentin is a limitation of this study, as it differs both in structure and composition from human dentin.^39^ Furthermore, root and crown dentin may also vary in different properties affecting experimental outcomes. However, due to reasons of availability and an existing inhomogenity also within human dentin, preference was given to bovine dentin. Its comparability was proven by Wegehaupt et al^56,57^ for both abrasion and erosion studies. Bovine dentin has also been used in several previous studies.^18–25,32,47,48,56,57^ Although for the average person with healthy oral conditions, toothbrushing is usually performed on enamel, the use of dentin can be justified by enabling a comparison of the results to the ones from previous abrasion studies.^19,20,22–25,32,47,48,56,57^ Moreover, the aim of this study was not to quantitatively specify cleaning efficacy, but to compare the cleaning efficacy resulting from two different toothbrushes at different brushing forces.

The tested toothbrushes with a flat-trim brush head and 43 parallel tufts with approximately 40 round-end filaments per tuft largely correspond to the manual reference toothbrush by the American Dental Association (ADA), which has an additional row of four tufts.^8,51^ However, other types of toothbrushes with different specifications were shown to achieve better results both in terms of removing dental plaque and improving gingival inflammation indices.^13,46,64^ Therefore, it is questionable whether the ADA reference toothbrush can be seen as the toothbrush of choice in terms of personal oral care. There is a large and growing number of different toothbrushes available on the market, which makes it more and more difficult to give any universally valid recommendation applicable for all patients, especially considering the enormous diversity of oral conditions patients present with. To further reinforce oral hygiene practice, a personal consultation with individual instructions for the use of mechanical and chemical measurements is necessary.^26,27^ However, experimental and comparative reasons on an in-vitro level justify the use of these custom-made toothbrushes, as this study focuses on the isolated effects of its bristle stiffnesses and brushing force.

It must be mentioned that the staining protocol described does not imitate a realistic process of discolouration in vivo. The duration of 17 h of continuous staining is not comparable to the durations while drinking. In addition, the acidity of the solution could erode the dentin to a non-negligible extent, leaving it more porous and receptive for colour particles. Nevertheless, for this study, extensive staining was desired to facilitate comparability.

Abrasion during the process of toothbrushing critically depends on the toothpaste used.^61^ Many manufacturers declare the abrasivity of their toothpastes by providing RDA values (relative dentin abrasivity), with higher values generally indicating more abrasion on dentin.^40^ The lab-determined RDA of 67 of the slurry used here matches the range of most conventional toothpastes on the market, which ranges from about 30 to 100.^18^ However, it is not permissible to assume a direct relationship between abrasion on dentin and abrasion on enamel.^11,18^ Current studies investigating the correlation between the RDA or the more rarely used REA and the corresponding cleaning efficacy on dentin show opposing results.^44,48,63^ Furthermore, the interaction between RDA/REA and the cleaning efficacy on enamel has not yet been sufficiently evaluated.

Brushing forces of 1 – 4 N were chosen according to proven test protocols and recommended as well as actually applied forces during toothbrushing in-vivo by non-instructed adults (2 N and 2.3 ± 0.7 N at a maximum of 4.1 N respectively).^14^ In-vivo data by Ganss et al^14^ show a predominant brushing frequency of twice a day and a mean duration of 96.6 ± 36 s, which approximately fits general recommendations. Taking this into account, the shorter brushing time of 2 min was set. The brushing time of 25 min was chosen for the sake of comparability with a previous study performed by Hamza et al,^24^ where dentin abrasion was investigated under identical lab conditions, except for RDA values. The results measured in this study do not allow drawing a direct conclusion about the cleaning efficacy over time in vivo, as it is not only performed mostly on enamel, but is also influenced by great variability in brushing technique, number of strokes, distribution of brushing on different groups and surface areas of teeth, dilution of slurry, degree of wear of both teeth and toothbrush, etc, which cannot be recreated under laboratory conditions.

Compared to preceding studies on cleaning efficacy, the planimetrical analysis of the pictures taken with default settings could be improved by introducing a new way of digitalisation and evaluation. Through direct processing of the marked areas by the programme used, the percentage of clean surface area was directly disclosed, skipping the steps of manual capturing and digitalisation and therefore reducing the potential sources of error. The rating of cleanliness of the surface examined remains subjective and dependent on the viewer’s perception. This can be influenced and potentially distorted by different factors, such as variation in the discolouration patterns of the dentin samples, camera settings, light exposure, brightness and colour saturation of the display, as well as the individual’s colour vision or daily form of the investigator. There is potential for further improvements in defining and assessing clean surface areas. A purely digital assessment of tooth colour (lightness, chroma and shade) may be conceivable, e.g. with a spectrophotometer.

Consistent with the general consensus, increasing the brushing duration from 2 min to 25 min resulted in higher cleaning efficacy for both the soft- and medium-bristle toothbrush. The soft- and medium-bristle toothbrushes showed comparable values of cleaning efficacy after 2 min of brushing for all applied brushing forces (1 – 4 N), with only a statistically significantly lower value for 1 N using the medium-bristle toothbrush. After 25 min of brushing, the percentage of ‘clean’ surface area increased with increasing brushing forces for both the soft- and the medium-bristle toothbrush, with no statistically significant differences between the two stiffnesses. Together with the measured amount of dentin wear in the study by Hamza et al,^24^ this would support the postulate of positive correlation between dental wear and cleaning efficacy, at least for 1 N to 3 N of applied brushing force. At 4 N however, an uncoupling between abrasion and cleaning efficacy could be observed. While the dentin wear did not change statistically significantly for the medium-bristle brush and even decreased for the soft-bristle toothbrush, the increase in cleaning efficacy was still ongoing for both toothbrushes. Therefore, abrasion is not likely to be the only determinant of cleaning efficacy, although it seems to play an important role. Hamza et al^24^ supposed that some additional ingredients such as bleaching agents or blue-light filters could remove staining without mechanical means, or simply mask them. This could lead to better results in cleaning efficacy while at the same time minimising dental wear. The differences in the RDA values of the present study (RDA 67) and the previous paper (RDA 121) must be taken into consideration. One can assume that due to the considerably lower RDA of the slurry used in the present study, 25 min of brushing would result in lower abrasion compared to the same process with a slurry of higher abrasivity. However, the link between RDA and dentin wear is not likely to be directly proportional.^23,44,63^ It would therefore be interesting to compare results for both abrasion and cleaning efficacy, brushed with a slurry of the same RDA value.

The two types of toothbrushes used in this study only differed in the length of their bristles which at the same time defined their bristle stiffness. It is therefore conceivable that during brushing, the longer bristles of the soft toothbrush tend to bend to a greater extent than the shorter ones from medium bristle toothbrush, especially at higher brushing forces applied. This might have a crucial influence on the abrasive process on dentin. While the exact mechanism remains speculative, it is imaginable that after toothbrushing, the surface is left with a different texture. Different brushing angles and contact areas with which the bristles process the samples, especially at higher loads, might be provide an explanation. Tawakoli et al^48^ demonstrated abrasive potential to statistically significantly correlate with surface roughness as well as cleaning efficacy. Although surface roughness was not evaluated in this study, it might deliver important information about the interplay between abrasion, surface texture and perceived cleanliness. In order to develop a better understanding of how cleaning efficacy comes about, further investigations are desirable.

## Conclusion

This study showed that soft- and medium-bristle toothbrushes (defined as such only according to their bristle length) provide comparable cleaning efficacy independently of the brushing force applied and duration of brushing. While the brushing force had almost no influence during short brushing periods (2 min), cleaning efficacy increased with increasing brushing forces during longer brushing (25 min).
